# Functional Analyses of House Fly Carboxylesterases Involved in Insecticide Resistance

**DOI:** 10.3389/fphys.2020.595009

**Published:** 2020-10-16

**Authors:** Xuechun Feng, Nannan Liu

**Affiliations:** ^1^Department of Entomology and Plant Pathology, Auburn University, Auburn, AL, United States; ^2^Division of Biological Sciences, University of California, San Diego, San Diego, CA, United States

**Keywords:** carboxylesterases, insecticide resistance, *Musca domestica*, *in vitro* metabolism, MTT assay, homology modeling and docking analysis

## Abstract

Carboxylesterase-mediated metabolism is one of major mechanisms involved in insecticide resistance. Our previous study has identified multiple carboxylesterase genes with their expression levels were significantly upregulated in pyrethroid resistant house flies. To further explore their metabolic functions, we used insect *Spodoptera frugiperda* (*Sf*9) cells to express these carboxylesterases *in vitro* and measure their hydrolytic activities toward esterase substrates. Our results indicated that these carboxylesterases can efficiently hydrolyze α-naphthyl acetate rather than β- naphthyl acetate. A cell based MTT cytotoxicity assay indicated that carboxylesterase-expressing cells show enhanced tolerance to permethrin, suggesting important roles of these carboxylesterases in metabolizing permethrin and thereby protecting cells from permethrin treatments. The metabolic functions of carboxylesterases were further verified by conducting *in vitro* metabolism studies toward permethrin and its potential metabolites 3-phenoxybenzyl alcohol and 3-phenoxybenzaldehyde, which not only suggested the potential metabolic pathway of permethrin in insects, but also important roles of these candidate carboxylesterases in metabolizing permethrin and conferring resistance in house flies. Homology modeling and docking were finally conducted to reflect interactions between permethrin ligand and carboxylesterase proteins, visually confirming the metabolic functions of carboxylesterases to insecticides in house flies.

## Introduction

House flies, *Musca domestica*, are ubiquitous agricultural and sanitary pests that can mechanically transmit more than 100 human and animal disease pathogens, including bacterial, protozoan, helminthic, and viral pathogens (Sasaki et al., 2000; Barin et al., 2010; Gordon Hewitt, 2011; Abbas et al., 2014; Scott et al., 2014). Pyrethroids are currently the most widely used insecticides for the management of many different insects including house flies due to their high insecticidal potency, low mammal toxicity and environmental friendliness (Casida et al., 1983; Soderlund and Knipple, 2003). However, house flies can rapidly develop resistance and cross-resistance to insecticides, which is a major concern for house fly control strategies worldwide (Liu and Yue, 2000; Kaufman et al., 2010; Scott et al., 2013; Abbas et al., 2014).

Previous work to characterize the underlying molecular basis for the development of insecticide resistance has already laid the foundation for a better understanding of this important issue and facilitated efforts to design novel strategies to efficiently prevent or minimize the spread and evolution of resistance development in many insect pests (Hemingway and Ranson, 2000; Roush and Tabashnik, 2012). The interactions of multiple mechanisms (i.e., increased detoxification and decreased target site sensitivity) or genes (i.e., cytochrome P450s and carboxylesterases) responsible for insecticide resistance have been extensively studied in recent years (Corbel et al., 2007; Bass et al., 2014; Liu, 2015). In particular, carboxylesterases, as one of the major detoxifying enzymes in insects, have attracted attentions for their potential role in sequestering and metabolizing insecticides (Wheelock et al., 2005; Grigoraki et al., 2015; Grigoraki et al., 2017). Multiple carboxylesterase genes have shown to be transcriptionally up-regulated in various resistant insects, including house flies (Cao et al., 2008; Bao et al., 2010; Zhang et al., 2010; Adelman et al., 2011; Bass and Field, 2011; Fuentes-Contreras et al., 2013; Demkovich et al., 2015). These overexpressed carboxylesterases are thought to sequester the insecticides and hydrolyze them into less harmful substances, thus facilitating excretion outside the insect bodies (Field and Blackman, 2003; Wheelock et al., 2005). In both *Aedes aegypti* and *Anopheles gambiae* mosquitoes, pyrethroids can be metabolized by carboxylesterases to form PBOH (phenoxybenzoic alcohol) and PBCHO (phenoxybenzaldehyde), which can be further metabolized by cytochrome P450 monooxygenases to PBCOOH (phenoxybenzoic acid) (Somwang et al., 2011; Chandor-Proust et al., 2013).

Our previous studies explored expression profiles of all carboxylesterases in different house fly strains and revealed four carboxylesterase genes, *Md*αE7, *Md*βE2, *Md*αE17, and *Md*IntE7, with their expressions of 3- to 15-fold higher in pyrethroid resistant house fly strain compared to those in susceptible house fly strains, indicating the potential involvements of these carboxylesterases in insecticide resistance of house flies (Feng et al., 2018). To further characterize the metabolic roles of carboxylesterase against insecticides, we investigated the functions of *Md*αE7 in metabolizing permethrin *via* MTT cytotoxicity assay and *in vitro* metabolism assay, revealing high efficiency of *Md*αE7 against permethrin in different ways (Feng and Liu, 2018). We hypothesized that the development of insecticide resistance was conferred by the combination of multiple overexpressed metabolic genes in house flies. To test our hypothesis, in the current study, we functional characterized the up-regulated carboxylesterase genes through *in vitro* metabolism studies, insect cell viability measurements, and homology modeling and docking analysis. Our study provided direct evidence for the involvement of multiple carboxylesterases in metabolizing permethrin, resulting in pyrethroid resistance in house flies.

## Materials and Methods

### The House Fly Strain

ALHF, a multi-insecticide resistant house fly strain, was used in this study. ALHF was collected from a poultry farm in Alabama in 1998 (Liu and Yue, 2000). This strain was further selected with permethrin in the laboratory for six generations after collection and maintained under biannual selection with permethrin, reaching to a high resistance level (∼2000 fold) to permethrin (Liu and Yue, 2000; Tian et al., 2011; Feng et al., 2018). The house flies were reared at 25 ± 2°C under a photoperiod of 12:12 (L: D) hours and fed with sugar and water.

### Construction of pENTR^TM^ Expression Plasmids of Carboxylesterase Genes

The total RNA was extracted from 20 3-day old adult female ALHF house flies using the acidic guanidine thiocyanate-phenol-chloroform method (Chomczynski and Sacchi, 2006). The DNA was removed from the total RNA using DNase (TURBO DNA-free, Ambion). The first-strand cDNA was synthesized with DNA-free total RNA using a Transcriptor First Strand cDNA Synthesis Kit (Roche) and oligo-dT primer following the manufacturer’s instructions. The pENTR^TM^ expression plasmids of carboxylesterases were constructed with gene-specific primers designed based on their full-length nucleotide sequences^1^ with four nucleotide bases CACC added to the 5′ end of forward primer (immediately upstream of the ATG transcription start codon) (Supplementary Table 1), which enables the carboxylesterase genes to be directly cloned into the pENTR^TM^ TOPO^®^ vector (Invitrogen) by annealing the CACC sequence in the PCR products with the overhang tag GTGG in the vector. The GFP gene was amplified from a plasmid kindly provided as a gift, with CACC added to allow the construction of pENTR^TM^ expression plasmid (Supplementary Table 1). The recombinant vector was then transformed into One Shot^®^ competent *E. coli*. pENTR^TM^ plasmids with target carboxylesterase genes were purified using the PureLink HQ Mini plasmid purification Kit (Invitrogen). The orientation of the inserted genes was detected by using the forward primer of each of the specific genes and the reverse primer of M13. Expression plasmids were further verified by sequencing.

### Recombinant Baculovirus Expression of Carboxylesterases in Sf9 Cells

The detailed methods follow our previous protocol (Feng and Liu, 2018). Briefly, the pENTR^TM^ plasmid of each carboxylesterase gene and GFP gene was ligated with BaculoDirect Linear DNA using the LR clonase^TM^ II enzyme mix through the BaculoDirect^TM^ Baculovirus Expression System. The constructed recombinant baculovirus was then transfected into *Spodoptera frugiperda* (*Sf*9) cells using Cellfectin II Reagent (Invitrogen) to produce recombinant baculovirus stock solutions. The large-scale expression of carboxylesterase proteins in the *Sf*9 cells was performed according to the manufacturer’s instructions (Invitrogen). The titer of the baculovirus was measured by plaque forming assay and a titer of ∼2 × 10^8^ pfu/mL P2 virus was used to infect *Sf*9 cells for large-scale expression of carboxylesterase proteins, largely because the cells own the highest infection ratio at the same time with lowest cell death ratio under P2 infection stage compared with at other infection stages (Feng and Liu, 2018). The parental *Sf*9 cells and pENTR^TM^ CAT (plasmid producing baculovirus expressing chloramphenicol acetyltransferase (CAT) protein [Invitrogen]) infected cells served as controls. The cell lysate protein was harvested after 72 h infection and centrifuged at 1000 rmp for 10 min at 4°C. The cell pellets were washed twice using ice-cold PBS buffer (pH 7.4) and then re-suspended in insect cell PE LB^TM^ buffer. Subsequently, the dissolved cell lysate was centrifuged at 9800 rmp for 15 min, and then the supernatant was collected and stored at −80°C. The protein concentration was measured with Bradford method (Bradford, 1976). For each protein, three replications for individual infection were performed to produce different batch of carboxylesterase proteins.

### Carboxylesterase Activity Assays

The activities of the carboxylesterases were determined by measuring their hydrolysis toward α-naphthyl acetate (α-NA) and β-naphthyl acetate (β-NA), both of which are ester substrates commonly used for esterase activities. The method follows Zhang et al. (2010) with modifications based on our pretests, in which we found that the protein amount around 50 μg is adequate for measuring the hydrolytic activities and meet the detection limit of the spectrophotometer. Briefly, a 25 μL of carboxylesterase protein solution (with a final concentration of 2.0 μg/μL protein in 0.1 M PBS buffer, pH 7.5) and 90 μL of 3 × 10^–4^ M substrate solution (either α-NA or β-NA dissolved in 0.1 M PBS buffer) were added to a 96-well microplate and incubated at 30°C for 30 min. The reaction was stopped by the addition of 45 μL of freshly prepared diazo blue-sodium lauryl sulphate solution (containing 2 parts of 1% fast blue B salt and 5 parts of 5% sodium dodecyl sulfate solution) in each well. After 15 min incubation at room temperature, the absorbance value of hydrolysis product α-naphthol or β-naphthol was measured at 600 or 550 nm, respectively, with a 96-well microplate reader (Cytation 3 imagine reader, BioTek, United States) and then converted to product formation rate (pmol/min/mg protein) based on the standard curves for α-naphthol or β-naphthol. For the kinetic assay, we conducted pretest for the range of substrate concentration (0.01 to 2 mM) before the real experiments and showed that the substrate concentration at a range of 0.1 to 1.2 mM had the better degradation curve by enzymes and met the detection limits of the spectrophotometer reader. Accordingly, the kinetic parameters, including Michaelis constant (Km) and maximal velocities (Vmax) for each carboxylesterase were measured using a series of substrate (α-NA or β-NA) concentrations ranging from 0.1 to 1.2 mM (add 3.8, 7.7, 11.5, 15.3, 19.2, 23.0, 26.8, 30.7, 34.5, 38.3, 42.2, and 46.0 μL of 3 × 10^–3^ M substrate solution, respectively, into each well). The reactions with proteins extracted from the parental Sf9 cells or CAT-recombinant baculovirus infected cells served as controls. Three replications were performed with independent prepared proteins.

### MTT Cytotoxicity Assay

The cytotoxicity assay of the carboxylesterases was conducted according to Gong et al. (2017) with modifications. The cell infection procedures follow methods described previously (Feng and Liu, 2018). The cells at P2 infected stage (with a titer of ∼2 × 10^8^ pfu/mL) were cultured in 25 cm^2^ flasks at 27°C. The pENTR^TM^ CAT expressing cells cultured under the same conditions was served as controls. After 48 h cultivation, cells expressing either GFP or different carboxylesterases were seeded onto 24 well plates with a density of 2 × 10^5^ cells/well, and later treated with permethrin standard solutions (the mixture of *cis-* and *trans-*isomers dissolved in acetonitrile) (analytical standard, Sigma-Aldrich), with final concentrations ranging from 50 to 400 μM. The cytotoxic effects of the permethrin standards were evaluated with a MTT cell viability assay kit (Sigma). After 48 h treatment, the cell culture medium was removed and the cells were washed with PBS buffer (0.1 M, pH 7.4). Later, 200 μL of thiazolyl blue tetrazolium bromide solution (Sigma-Aldrich) (5 mg/mL) was added to each well and the plate was incubated at 37°C for 4 h, after which the absorbance values were measured at 540 nm using the Cytation 3 imagine reader (BioTek, United States). Four replications were conducted with independent protein preparations. The cell viability was calculated in comparison with acetonitrile-treated cells. For the inhibition assay, the inhibitor S, S, S-tributyl phosphorotrithioate (DEF) (Sigma-Aldrich) (with final concentrations of 0.1, 1, and 10 μM) was added, together with 200 μM permethrin in each well, and cell viability was calculated in comparison with treatments with no DEF added.

### *In vitro* Metabolism of Permethrin and Its Metabolites by Carboxylesterases

Each substrate standard (Permethrin, PBOH or PBCHO) was initially dissolved in acetonitrile to make 1 mM stock solution. Serial dilutions of stock solution were then prepared in acetonitrile to create the standard curve for each. A total of 700 μL metabolism reaction contained 20 μM substrate standard and 1 mg carboxylesterase protein (*Md*αE17, *Md*βE2 or *Md*IntE7) dissolved in 0.2 M Tris–HCl buffer. After 2 h incubation at 30°C with orbital shaking, the reaction was quenched by adding 700 μL ice-cold acetonitrile and incubated with shaking for an additional 15 min. After that, the mixture was centrifuged at 10000 rmp for 2 min and the supernatant was collected by filtering through 0.45 μm membranes and transferred to ultraclean glass vials for HPLC analysis. The HPLC analysis was monitored by a reverse-phase HPLC system (Alliance Waters 2695) equipped with a Nova-Pak C18 column (60 Å, 4 μm, 3.9 mm × 150 mm, 1/pkg [WAT086344]) and a Waters 2487 Dual λ absorbance detector. Two mobile phases (mobile phase A: 90% acetonitrile and 10% water; mobile phase B: 5% acetonitrile adjusted to pH 2.3 with 85% phosphoric acid) were used for the gradient elution with a flow rate of 1 ml/min and measured at a wavelength of 232 nm. The gradient system was initiated with 50% of solvent A and 50% of solvent B rising to 75% of mobile phase A at 6 min and finishing at 100% of solvent A at 8 min. The flow of 100% mobile phase A was maintained for 4 min and then reduced to 50% at 13 min and continued for a further 4 min to return the column to the initial conditions for the next run. Reactions containing no enzymes were used to calculate the substrate depletion percentage. Three replications were performed, and a paired *t*-test was used to analyze the results. Reactions with proteins extracted from CAT expressing cells served as controls.

### *In silico* Modeling and Docking Analysis

*In silico* 3D structure modeling of each carboxylesterase protein was performed by the I-TASSER server utilizing the combined methods of threading and *ab initio* modeling^2^ (Roy et al., 2010; Zhang et al., 2011). Five models were predicted for each carboxylesterase gene and the top scoring model submitted to the FG-MD server for fragment guided molecular dynamics structure refinement (Zhang et al., 2011). Model quality was controlled by Ramachandran plots generated with Procheck^3^ (Laskowski et al., 1993) and ProSA-web^4^ (Sippl, 1993; Wiederstein and Sippl, 2007). Proteins and ligands were prepared for docking with Autodock Tools V1.5.6^5^. Molecular docking was performed by Autodock 4.2 (Morris et al., 2009). Ligand permethrin structures were retrieved from the ZINC database^6^ (Irwin and Shoichet, 2005). For all dockings, a search space with a grid box of 60 × 60 × 60 Å centered on the serine of the catalytic triad of the carboxylesterase was used. All protein structure images were produced by Pymol^7^ (DeLano, 2002). The binding cavity and its constitutive amino acids were predicted by LigPlot (Wallace et al., 1995). Protein structure diagrams were produced using TopDraw (Bond, 2003).

## Results

### Carboxylesterase Activity

The expression of carboxylesterase protein was accomplished by infecting insect *Sf*9 cells with constructed carboxylesterase-recombinant baculovirus. The carboxylesterase proteins obtained were isolated from *Sf*9 cells and further used for biochemical characterization. Our results indicated that carboxylesterase proteins obtained from insect *Sf*9 cells were capable of hydrolyzing α-naphthyl acetate (α-NA) to produce α-naphthol at different efficiencies, with a hydrolytic activity ranging from 6083.5 to 13810.1 pmol⋅min^–1^⋅mg^–1^, which were 1.8–4.0 fold higher than that measured in either the parental *Sf*9 cells or the pENTR^TM^ CAT (plasmid producing baculovirus expressing chloramphenicol acetyltransferase (CAT) protein [Invitrogen]) infected cells served as controls (Table 1), indicating the strong hydrolytic capabilities of carboxylesterases in metabolizing esterase substrate α-NA. The kinetic parameter Km and Vmax for each hydrolytic reaction were also measured by using substrate α-NA with final concentrations from 0.1 to 1.2 mM and listed in Table 1. These data indicated that all these reactions followed the Michaelis-Menten equation; The maximum velocities (Vmax) of carboxylesterase reactions were ranging from 41048.9 to 71586.7 pmol⋅min^–1^⋅mg^–1^, significantly higher than the values in controls (9503.9 pmol⋅min^–1^⋅mg^–1^ for parental Sf9 cells and 10343.6 pmol⋅min^–1^⋅mg^–1^ for pENTR^TM^ CAT infected cells, respectively), suggesting the strong hydrolytic capabilities of these carboxylesterases toward substrate α-NA. Simultaneously, we examined the hydrolytic activities and kinetic parameters of different carboxylesterases to another substrate, β-naphthyl acetate (β-NA), and the hydrolytic values are low (less than 20.0 pmol⋅min^–1^⋅mg^–1^) in all these genes (Table 1).

**TABLE 1:** Hydrolytic activities and kinetic parameters of *M. domestica* carboxylesterases.

Enzyme	α-naphthyl acetate			β-naphthyl acetate		
	Activity^*a*^	Km^*b*^	Vmax^*c*^	Activity^*a*^	Km^*b*^	Vmax^*c*^
Sf9 cells	3458.2 ± 168.2	186.4 ± 7.6	9503.9 ± 151.0	8.4 ± 0.5	56.4 ± 3.0	9.9 ± 0.2
CAT gene	3645.0 ± 173.5	213.2 ± 10.7	10343.6 ± 186.8	8.8 ± 0.6	58.1 ± 3.0	9.8 ± 0.1
*Md*αE7	11396.6 ± 484.8	568.0 ± 22.3	53555.5 ± 1649.7	12.9 ± 0.6	146.8 ± 4.1	15.7 ± 0.3
*Md*αE17	13810.1 ± 635.6	651.9 ± 33.8	71586.7 ± 2585.8	18.4 ± 1.7	190.1 ± 4.3	21.9 ± 0.7
*Md*βE2	8610.8 ± 335.2	523.3 ± 19.5	46414.3 ± 1401.9	13.8 ± 1.0	188.3 ± 3.5	16.0 ± 0.3
*Md*IntE7	6083.5 ± 326.6	521.7 ± 15.4	41048.9 ± 1748.2	11.5 ± 0.6	146.1 ± 4.8	13.9 ± 0.1

*All data were listed as mean ± STEa: COE activity = pmol⋅min^–1^⋅mg^–1^. b: Km (Michaelis constant) = μM. c: Vmax (Maximum velocity) = pmol⋅min^–1^⋅mg^–1^.*

### Cytotoxicity of Permethrin in Carboxylesterase-Expressing Sf9 Cells (MTT Assays)

**FIGURE 1:**
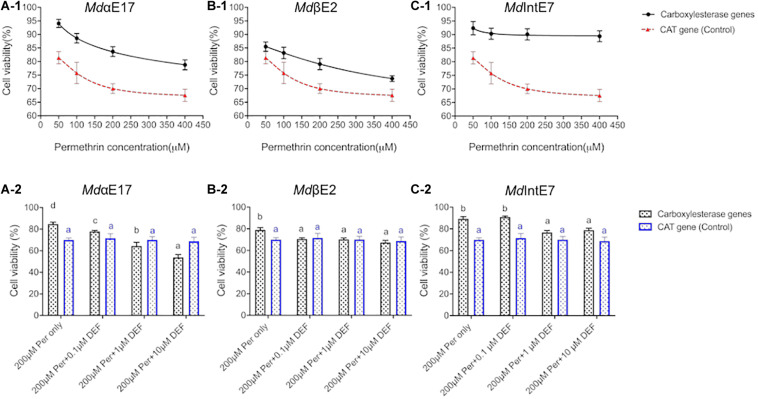
Roles of carboxylesterases in detoxification of permethrin in insect *Sf*9 cells. **(A-1)**, **(B-1)**, and **(C-1)** The comparisons of *Sf*9 cell viabilities expressing *Md*αE17, *Md*βE2, and *Md*IntE7 to *Sf*9 cell viabilities expressing CAT gene (control), respectively, treated with 50, 100, 200, and 400 μM of permethrin. **(A-2)**, **(B-2)**, and **(C-2)**. The comparisons of *Sf*9 cell viabilities expressing *Md*αE17, *Md*βE2, and *Md*IntE7 to *Sf*9 cell viabilities expressing CAT gene (control), co-treated with 200 μM permethrin and 0.1, 1, or 10 μM of DEF. Three replications with independent prepared proteins were performed. Student’s *t*-test was used for the statistical significance analysis. Different letters above the bars indicate the significant differences of cell viabilities under different treatments (*P* ≤ 0.05). The blue color line and shape indicated the control groups (CAT-infected cells).

To confirm that *Sf*9 cells can be applied to study *in vivo* metabolic functions of carboxylesterases, and to prove that MTT assay is a feasible method to measure cell viabilities against different permethrin stimuli, we firstly build a GFP-recombinant baculovirus and use it to infect insect *Sf*9 cells. The GFP expression make it visible to directly observe cell viabilities under different conditions. The 48 h-post infection cells at P2 stage were then chosen to perform the permethrin-treated experiments due to the relative higher cell viability and protein expression levels at this stage (Feng and Liu, 2018). By treating the GFP-expressing cells with permethrin at different concentrations of 50, 100, 200, and 400 μM, we observed that cell viabilities were gradually decreased as the increment of permethrin concentrations (Supplementary Figure 1) and no significant differences were observed in two controls (one is cells without permethrin treatments and the other with only acetonitrile added) (Supplementary Figure 1), suggesting that the permethrin rather than the virus itself can greatly influence the cell viability. Also, no significant differences were observed in two controls (one is cells without permethrin treatments and the other with only acetonitrile added) (Supplementary Figure 1), indicating that acetonitrile used to dissolve permethrin will not affect cell viabilities. We then use the same method to test the cytotoxicity of permethrin among all carboxylesterases and cell viabilities against different permethrin concentrations (50, 100, 200, and 400 μM) was calculated in comparison with cells treated with acetonitrile alone. The pENTR^TM^ CAT expression Sf9 cells was used as the controls. Our results showed that the viabilities of *Md*αE17 expressing cells were significantly higher (78.8–94.1%) than those of controls, which were 57.7–88.9% (Figure1A-1) when exposed to permethrin at different concentrations. The cell viabilities of *Md*IntE7 expressing cells ranged from 89.4 to 92.4%, again more tolerant than the controls (Figure 1C-1); the cell viabilities of *Md*βE2 expressing cells, though weaker than those of *Md*αE17 and *Md*IntE7, were still significantly higher than the controls, ranging from 73.3 to 85.5% (Figure 1B-1). The results show that carboxylesterase-expressing cells can greatly enhance their tolerance to permethrin treatments compared with control cells.

**FIGURE 2:**
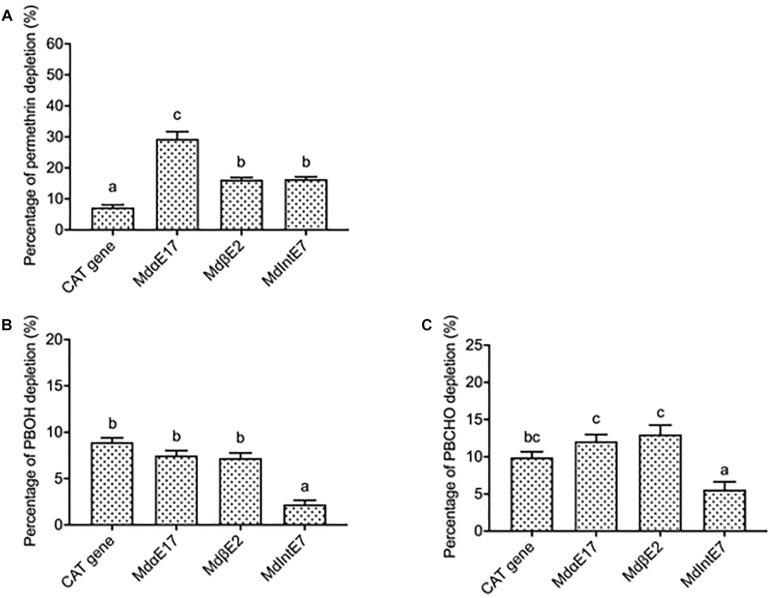
The depletion percentages of permethrin, PBOH and PBCHO by different carboxylesterases. **(A)** The depletion percentages of permethrin by carboxylesterases. **(B)** The depletion percentages of PBOH by carboxylesterases. **(C)** The depletion percentages of PBCHO by carboxylesterases. Three replications with independent prepared proteins were performed. Different letters above the bars indicate the significant differences for the depletion percentage of substrates under different treatments (*P* ≤ 0.05).

To deeper explore roles of these carboxylesterases in detoxifying permethrin in insect cells, we then tested the cell viabilities against permethrin in presence of the carboxylesterase inhibitor, S, S, S-tributyl phosphorotrithioate (DEF). The cell viabilities in *Md*αE17 expressing cells were significantly dropped from 84.7% (200 μM permethrin treatment in absence of DEF) to 77.8, 64.4, and 53.8% when co-treated with 0.1, 1, or 10 μM DEF, respectively (Figure 1A-2). For *Md*βE2, a slight decrease in cell viability was detected between the control (79.1%) and the cells subjected to co-treatment with 200 μM permethrin and 0.1, 1 or 10 μM DEF (70.5, 70.2, and 67.4%, respectively) (Figure 1B-2). For *Md*IntE7, although no significant difference was detected between the control (89.4%) and cells co-treated with 200 μM permethrin and 0.1 μM DEF (91.0%), significant decreases to 76.8 and 78.9% were observed when co-treated with 200 μM permethrin and 1 or 10 μM DEF, respectively (Figure 1C-2). No significant differences in the cell viabilities were detected in pENTR CAT infected cells when co-treated with 200 μM permethrin and DEF at different concentrations. The significant decrease in cell viability against permethrin cytotoxicity in carboxylesterase expressing cells when co-treated with DEF at different concentrations strongly supports the involvement of these carboxylesterases in metabolizing permethrin in insect cells.

### *In vitro* Metabolism of Permethrin, PBOH and PBCHO by Carboxylesterases

Permethrin metabolism was assayed by incubating a 20 μM permethrin standard together with different carboxylesterase proteins extracted from infected Sf9 cells. The reactions of 20 μM permethrin incubated with proteins isolated from pENTR^TM^ CAT infected cells was served as the control. The depletion percentage for permethrin was calculated in comparison with reactions where only 20 μM permethrin was added. Reactions were monitored by reverse-phase HPLC after a 120 min incubation period. Since the permethrin standard is actually a mixture of *cis-* and *trans-* isomers, two peaks were observed in the HPLC chromatographic profiles, with elution times of 10.7 and 10.9 min for *trans-*permethrin and *cis-*permethrin, respectively (Supplementary Figure 2A). The highest depletion percentage of permethrin was achieved by *Md*αE17, at 29.4 ± 2.3% (Figure 2A), followed by 16.4 ± 0.7% and 16.2 ± 0.7% for *Md*IntE7 and *Md*βE2, respectively (Figure 2A). All depletion percentages for these carboxylesterases were significantly higher than that of pENTR^TM^ CAT gene (7.3 ± 0.8%) used as the control (Figure 2A).

The depletion percentages of two permethrin metabolites, PBOH and PBCHO, were also measured by incubating 20 μM substrate together with different carboxylesterase proteins extracted from insect *Sf*9 cells. The reaction of 20 μM substrates incubated with proteins extracted from pENTR^TM^ CAT infected cells again was served as the control. The depletion percentage of the substrate was again calculated in comparison with reactions in which only 20 μM substrates were added. Reactions were monitored by reverse-phase HPLC after a 120 min incubation period. The retention time of PBOH was 3.3 min (Supplementary Figure 2B). The depletion percentages of PBOH by *Md*αE17 and *Md*βE2 were 7.5 ± 0.5 and 7.2 ± 0.6%, respectively, none of which were significantly different from that of the pENTR^TM^ CAT infected cells (8.9 ± 0.5%) (Figure 2B). For *Md*IntE7, the depletion percentage of PBOH was 2.2 ± 0.5%, lower than that achieved by the control (Figure 2B). The retention time of the other substrate, PBCHO, was 5.4 min (Supplementary Figure 2C). Again, no significant differences were found in the depletion percentages of PBCHO achieved by *Md*αE17 (12.1 ± 1.00%) and *Md*βE2 (13.0 ± 1.2%) compared with that of the control (9.9 ± 0.8%) (Figure 2C).

### Homology Modeling and Permethrin Docking Analysis

**FIGURE 3:**
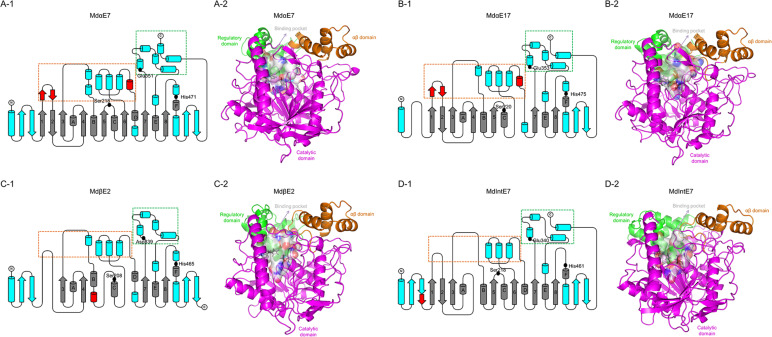
The structures of carboxylesterases in *M. domestica*. **(A-1)**, **(B-1)**, **(C-1)**, and **(D-1)** Topology representations of secondary structures of *Md*αE7, *Md*αE17, *Md*βE2, and *Md*IntE7 displaying the conserved α/β hydrolase fold (labeled as gray), conserved motifs among four carboxylesterase proteins (labeled as blue) and unique motifs belong to certain carboxylesterase structure (labeled as red). Two subdomains made up of bundles of α-helices (framed by orange and green box) formed the substrate binding cavity. Three conserved amino acids (Serine, Histidine, and Glutamine/Aspartic acid) consisted of a catalytic triad were also indicated as black dots. **(A-2)**, **(B-2)**, **(C-2)**, and **(D-2)** Cartoon representations of structures of *Md*αE7, *Md*αE17, *Md*βE2, and *Md*IntE7 highlighting the regulatory domain (green area), αβ domain (orange area), catalytic domain (magenta area) and binding pocket (white surface area). The conserved catalytic residues were also labeled as black sticks.

To investigate the interactions between carboxylesterases and permethrin, we conducted the homology modeling and permethrin docking analysis for four carboxylesterases. Several missing or inserted motifs were found in certain carboxylesterases, such as the missing of start antiparallel β-sheets, β6 and αD in *Md*αE17 (Figures 3B-1,B-2); the missing β1, β2, β5, and β6 and the insertion of a short helix following αB in *Md*βE2 (Figure 3C-1,C-2); and a missing β4 and the insertion of a short sheet after the start antiparallel β-sheets in *Md*IntE7 (Figure 3D-1,D-2). The overall structures containing eight-stranded β-sheet (β1–β8) surrounded by six α-helices (αA-αF), together with two pairs of antiparallel β-strands at the start and end of the protein structure, all of which were comprised of the catalytic domain (shown as the magenta area in Figure 3). Two bundles of α-helices at the top of the catalytic domain formed the αβ domain and the regulatory domain (shown as orange and green areas, respectively, in Figure 3). A catalytic triad made up of Ser, His, and Glu/Asp (labeled as black dots and sticks in Figure 3) was highly conserved among these four carboxylesterases. Two subdomains on either side of the active site cleft on the upper face of the protein formed the substrate binding cavity (white surface area in Figure 3). Subdomain I consisted of two short antiparallel α-helices inserted after β1, two short α-helices inserted after β3 and four α-helices following β6 (boxed orange in Figure 3). Subdomain II was composed of four α-helices inserted after β7 with the last two α-helices near the C-terminals (boxed green in Figure 3). A detailed comparison revealed that two small regions were divergent in the four carboxylesterase proteins, which could have a major impact on the rearrangement of the two subdomains that form the binding cavity. The first is the antiparallel β-sheet after β1 (shown as a red helix in Figures 3A-1,B-1), which is present in *Md*αE7 and *Md*αE17, while absent in *Md*βE2 and *Md*IntE7. This structure can create a groove against the N-terminal α-helices when packing, thereby preventing the partial closure of the active site. The second is the short helix before the αD (shown as red sheets in Figures 3A-1,B-1), which is present in *Md*αE7 and *Md*αE17 but absent in *Md*βE2 and *Md*IntE7. This helix is thought to hold apart two subdomains that comprise the binding cavity, leaving a much more open space for substrate binding.

**FIGURE 4:**
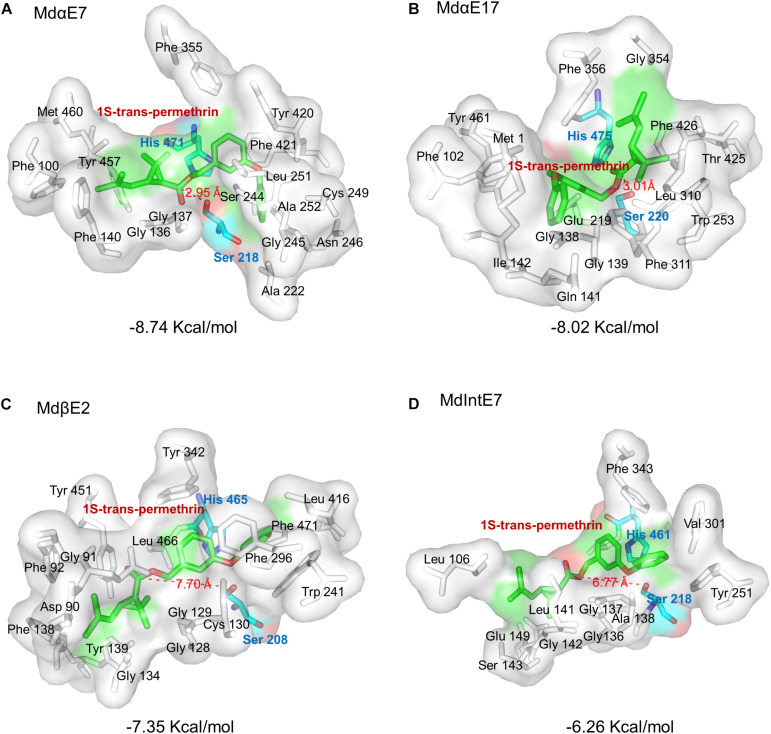
Stereo view of permethrin bound within the active site cavity. Permethrin bound within the active site cavity of: **(A)** *Md*αE7; **(B)** *Md*αE17; **(C)** *Md*βE2; and **(D)** *Md*IntE7. The Serine (Ser) and Histidine (His) residues are labeled as green sticks and the 1S-*trans-*permethrin isomer as cyan sticks. The distance between permethrin and the Ser residue of the carboxylesterase is indicated by a red dashed line. The binding energy is also shown. All amino acid active site cavities are labeled in each carboxylesterase structure.

Further docking analysis revealed the interactions between carboxylesterase proteins and permethrin. Our preliminary tests have individually docked four permethrin isomers into the carboxylesterases and found that 1S-*trans-*permethrin isoform fit most snugly into the binding pockets with lowest binding energy. We therefore chose 1S-*trans-*permethrin isomer for this analysis. From Figure 4, we observed the majority of constitutive amino acids forming binding cavities were hydrophobic, including Gly, Phe, Ala, Val, Leu, Ile, Met, and Trp, providing a hydrophobic environment for permethrin binding. The conserved catalytic triad composed of Ser, His, and Glu/Asp (shown in Figure 3) is involved in the catalytic process. Briefly, Ser residue attacks the carbonyl group of permethrin to form a tetrahedral intermediate, which then collapses to release Ser, the alcohol portion of permethrin and a acyl-enzyme complex, allowing for the His residue to further attack and release the acid portion of permethrin (Wheelock et al., 2005; Satoh and Hosokawa, 2006). Based on this, the distance between the oxygen atom in the OH side chain of Ser residue and the carbon atom in the carbonyl group of substrates is of great value to determine the distance between carboxylesterase and permethrin ligand. Our results in Figure 4 show that *Md*αE7 has the shortest distance (=2.95 Å) to permethrin with the lowest binding energy (=−8.74 Kcal/mol) (Figure 4A); The distance between *Md*αE17 and permethrin is 3.01 Å with a binding energy of 8.02 Kcal/mol (Figure 4B); both *Md*βE2 and *Md*IntE7 were even further from permethrin, at 7.70 and 6.77 Å and with relatively higher binding energies of −7.35 Kcal/mol and −6.26 Kcal/mol (Figures 4C,D), respectively.

## Discussion

In insects, carboxylesterase is one of the major metabolic enzymes that detoxify insecticides in the first phase of metabolism (Somwang et al., 2011; Chandor-Proust et al., 2013). Overexpressed carboxylesterases lead to increased activities, which further results in the enhanced metabolism of xenobiotics or endogenous compounds (Bass and Field, 2011; Zhang et al., 2013; Grigoraki et al., 2015). In a previous study we identified multiple carboxylesterase genes in house flies, whose expressions were not only constitutively up-regulated in the resistant house fly ALHF strain, but can also be induced to much higher levels in response to permethrin, indicating the important role they play in metabolizing permethrin in house flies (Feng et al., 2018). However, a functional characterization of these carboxylesterases *in vitro* is still lacking.

Here, a baculovirus-mediated insect *Sf*9 expression system was used to investigate the heterozygous expression of these carboxylesterases *in vitro*; their hydrolytic activities toward different esterase substrates and permethrin insecticides were also measured in this study.

Our results indicated that all carboxylesterases showed strong activities to α-NA rather than β-NA, indicating that β-NA may not be the most favorable substrate for measuring the activities of these carboxylesterases. The choice of a substrate with which to monitor carboxylesterase activity remains a major obstacle hampering efforts to accurately characterize carboxylesterase activities, especially for multiple isozymes (Wheelock et al., 2005). Also, all carboxylesterases significantly differ in their hydrolytic activities toward esterase substrates, which was partially attributed to the various protein structures leading to different substrate specificities (Hosokawa, 2008; Younus et al., 2017).

The data from GFP-expressing cells indicated that the MTT assay can be used to explore the cytotoxicity of permethrin for all carboxylesterases. Although its accuracy was sometimes limited by numerous experimental parameters correlated with cell metabolism and virus infections, but this can be diminished by performing more replicates with independent cell and virus preparations, and it is no doubt that MTT assay is still a rapid and easily operated method to quickly detected cell cytotoxicity against permethrin treatments, giving a preliminary images in consideration of metabolic roles of carboxylesterases in detoxifying permethrin in insect cells. Some other assays can compensate to more precisely measure and compare the metabolic efficiencies.

Compared with MTT assay, the *in vitro* metabolism study is recognized as the most accurate strategy to directly reflect the metabolic efficiencies of detoxifying enzymes against xenobiotics. In this study, the depletion percentages of permethrin and its substrates have been successfully measured via *in vitro* metabolism assays and in turn reflecting the metabolic efficiencies of different carboxylesterases. By comparing the metabolic data collected, we found that α-esterases, such as *Md*αE17 in this study and *Md*αE7 reported in previous study (Feng and Liu, 2018), have much higher metabolic abilities to permethrin compared with *Md*βE2 and *Md*IntE7, which is consistent with the metabolism and detoxification roles of the α-esterase clade reported in classification studies (Campbell et al., 2003; Flores et al., 2005; Wang et al., 2015; Feng and Liu, 2018). While the overall metabolic efficiencies of these carboxylesterases to permethrin (∼16 to ∼30%) are less efficient than those achieved by multiple CYP450s in mosquitoes (∼40−∼45%) (Gong et al., 2017), the carboxylesterases may serve as an “insecticide sink” that delays or prevents the interactions of insecticides and target sites rather than directly metabolizing them (Oakeshott et al., 2005; Li et al., 2007). Two main permethrin metabolites, PBOH and PBCHO, were also measured for their depletion percentages among these carboxylesterases, and no significant metabolic effects were detected, which can be explained by the hypothesized permethrin metabolic route proposed in mosquitoes, where carboxylesterases only play essential roles in the first phase of permethrin metabolism, and other enzymes such as cytochrome P450s or glutathione S-transferases are involved in following metabolism of permethrin metabolites (Somwang et al., 2011; Chandor-Proust et al., 2013).

The house fly *Md*αE7 is orthologous to blowfly *Lc*αE7, an α-esterase isolated from the Australian sheep blowfly *Lucilia cuprina* with its roles in organophosphate (OP) resistance in blow flies (Jackson et al., 2013). The metabolic functions of house fly *Md*αE7 towered insecticides (either OP or permethrin) have also been reported in several studies (Zhang et al., 2010; Feng and Liu, 2018). However, few studies have explored the interactions between carboxylesterases and insecticides through homology modeling and docking analysis. In this study, we built homology models for four carboxylesterases based on the crystal structure of *Lc*αE7. We compared their modeling and found that their overall structures are highly conserved with majority of αβ hydrolase structures, especially for the catalytic triad. The detailed comparison among their structures revealed several differences, and a small divergence in structures may significantly impact the overall topology of the substrate binding sites (Jackson et al., 2013). From our modeling, we can see *Md*αE7 and *Md*αE17 have much more open spaces in their active sites compared to *Md*βE2 and *Md*IntE7, allowing much more favored substrate binding and accommodations, which can be further linked with their relatively higher metabolic efficiencies against permethrin (Feng et al., 2018).

We further analyzed the binding modes of permethrin within active cavities of different carboxylesterases. The catalytic process of substrate within the carboxylesterases determines that the hydrogen bond distance as well as the binding energy are two important parameters to analyze the binding affinity. Usually, the lower binding energy together with a shorter distance between O residue of Ser and C in the carbonyl group of substrates indicate a stronger binding affinity between carboxylesterases and ligands. Among four carboxylesterase binding modes, the *Md*αE7 showed lowest binding energy as well as the shortest distance, which reflects its highest binding affinity to permethrin, which also provide evidence for its highest metabolic efficiencies against permethrin in this study. Also, through binding multiple permethrin isoforms in protein cavities, we realized that the stereochemistry is another important factor in esterase-mediated metabolism, and our docking analysis found that 1S-*trans-*permethrin had the best fit within binding cavities of four carboxylesterases, and the similar findings have also been reported in carboxylesterase E4 of the aphid *Myzus persicae*, which exhibits absolute specificity for hydrolyzed 1S-*trans-*permethrin rather than other isomers (Devonshire and Moores, 1982).

In conclusion, our study has firstly employed baculovirus-mediated insect cell expression system to express carboxylesterases and characterized their functions *in vitro* via different assays, which provided multiple choices for exploring metabolic functions of detoxifying proteins in insects. The results of these house fly carboxylesterases in metabolizing permethrin have further provided directed scientific evidence for the esterase-mediated resistance in insects that sheds light on the mechanisms governing insecticide resistance development and could lead to the development of innovative new pest management strategies.

## Data Availability Statement

The original contributions presented in the study are included in the article/Supplementary Material, further inquiries can be directed to the corresponding author.

## Author Contributions

NL and XF conceived and designed the study and wrote the manuscript. XF performed the experiments. NL prepared the materials. Both authors reviewed the manuscript.

## Conflict of Interest

The authors declare that the research was conducted in the absence of any commercial or financial relationships that could be construed as a potential conflict of interest.

## Footnotes

1 https://www.ncbi.nlm.nih.gov/genome/?term=Musca+domestica

2 http://zhanglab.ccmb.med.umich.edu/I-TASSER/

3 http://services.mbi.ucla.edu/SAVES/

4 http://prosa.services.came.sbg.ac.at/prosa.php

5 http://mgltools.scripps.edu/downloads

6 http://zinc.docking.org/

7 http://www.pymol.org/

## Supplementary Material

Supplementary Figure 1 The cell viabilities under different permethrin treatments. The GFP-expressing cells at P2 infection stage were observed under fluorescent microscope. As the increase of permethrin concentration, the cell viabilities were gradually decreased. The GFP-expressing cells without permethrin treatment and with only acetonitrile added were served as control groups.

Supplementary Figure 2 HPLC profiles of permethrin, PBOH and PBCHO. **(A)** The HPLC profile of permethrin standard (a mixture of trans-permethrin and cis-permethrin isomers). The red arrows indicate the peaks for the trans-permethrin and cis-permethrin isomers. **(B)** The HPLC profile of PBOH standard. The red arrow indicates the peak for PBOH. **(C)** The HPLC profile of PBCHO standard. The red arrow indicates the peak for PBCHO.

Supplementary Table 1 The primer list.
