# Supplementary figures and images for: Functional Analyses of House Fly Carboxylesterases Involved in Insecticide Resistance
Source: Front Physiol. 2020 Oct 16;11:595009. doi: 10.3389/fphys.2020.595009 (PMC7596742; doi:10.3389/fphys.2020.595009)

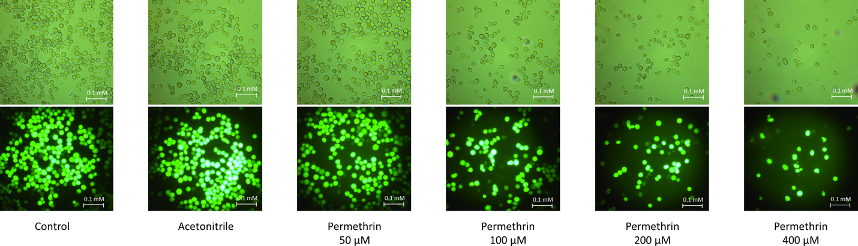

Supplement: Supplementary Figure 1 — The cell viabilities under different permethrin treatments. The GFP-expressing cells at P2 infection stage were observed under fluorescent microscope. As the increase of permethrin concentration, the cell viabilities were gradually decreased. The GFP-expressing cells without permethrin treatment and with only acetonitrile added were served as control groups. [file Image_1.png]

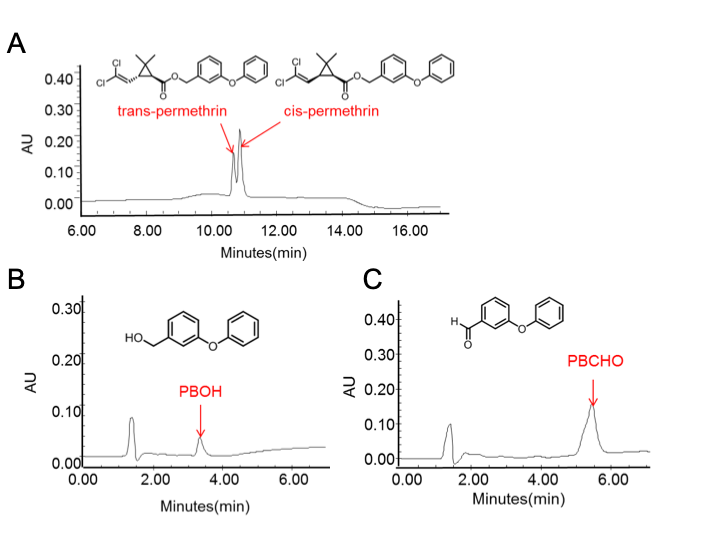

Supplement: Supplementary Figure 2 — HPLC profiles of permethrin, PBOH and PBCHO. (A) The HPLC profile of permethrin standard (a mixture of trans-permethrin and cis-permethrin isomers). The red arrows indicate the peaks for the trans-permethrin and cis-permethrin isomers. (B) The HPLC profile of PBOH standard. The red arrow indicates the peak for PBOH. (C) The HPLC profile of PBCHO standard. The red arrow indicates the peak for PBCHO. [file Image_2.TIFF]
