# Supplementary material for: Functional Analyses of House Fly Carboxylesterases Involved in Insecticide Resistance
Source: Front Physiol. 2020 Oct 16;11:595009. doi: 10.3389/fphys.2020.595009 (PMC7596742; doi:10.3389/fphys.2020.595009)
Supplement: Supplementary Table 1 — The primer list. [file Table_1.docx]

## Supplementary Table 1: The primer list

| Gene | Accession No. | Clade | Primers for Sf9 expression | |
| --- | --- | --- | --- | --- |
|  |  |  | Forward (5’-3’) | Reverse (5’-3’) |
| GFP | A plasmid gift from Dr. Park | | CACCATGGGCAGCAGCCATCATCATCA | TTAGCAGCCGGATCTCAGTGGTGGTGG |
| MdαE17 | XP_005175160 | α-esterase | CACCATGGATTTAAATATTGG | TTAACACAATGGCTCTTTG |
| MdβE2 | XP_005183940 | β-esterase | CACCATGAATTTCAAAGTTAG | TTAAAACAATTCCTTCTTTTTA |
| MdIntE7 | XP_005177448 | Integument esterase | CACCATGAAAGCATTGTGGTTC | TTAACTTAATTTCCAAATGCTTAACACT |
